# Food Intolerance After Bariatric Surgery: A Narrative Review of Prevalence, Mechanisms, and Dietary Management

**DOI:** 10.3390/nu17193118

**Published:** 2025-09-30

**Authors:** Karolina Brzostek, Iwona Boniecka

**Affiliations:** Department of Clinical Dietetics, Faculty of Health Sciences, Medical University of Warsaw, 01-445 Warsaw, Poland

**Keywords:** bariatric surgery, metabolic surgery, food intolerance, nutrition, sleeve gastrectomy, Roux-en-Y gastric bypass

## Abstract

**Background:** Bariatric surgery (BS) is the most effective long-term treatment for severe obesity, but many patients develop food intolerances that either reduce protein and micronutrient intake or shift consumption toward easily tolerated, calorie-dense “safe” foods (e.g., sweets, ice cream, sugar-sweetened beverages, refined-flour snacks), compromising nutrient adequacy and weight-loss maintenance. This narrative review summarizes evidence on the prevalence, mechanisms, and clinical impact of food intolerances after BS, focusing on red meat, dairy, water, cereal, and vegetables, and offers practical nutritional management strategies. **Methods:** A targeted literature search of PubMed and Cochrane Library from the past 10 years was conducted. Studies were selected based on relevance and quality. **Results:** Intolerance patterns vary by food type and surgical method. Red meat is the most frequently problematic food, with some patients symptomatic for years despite gradual improvement. Dairy products cause gastrointestinal discomfort in some cases, partly due to lactose intolerance. Cereal products may initially cause swallowing difficulties but are generally well tolerated two years postoperatively. Water intolerance mainly occurs shortly after sleeve gastrectomy (SG), linked to sleeve aperistalsis and edema, resolving within weeks. Cooked vegetables are overall well tolerated across procedures. Mechanisms behind intolerance include mechanical restriction, altered gastric emptying, exaggerated entero-hormonal responses, and new taste aversions. **Conclusions:** Food intolerances are a common, procedure-specific consequence of BS, requiring systematic assessment and personalized dietary management. Early management—modification of consistency, portion control, and supplementation—can improve product tolerance, prevent nutritional deficiencies, and support sustainable weight loss. However, further prospective studies on the mechanisms of food intolerances are needed.

## 1. Introduction

Morbid obesity is currently one of the most serious public health challenges, affecting over 890 million adults worldwide, with the upward trend persisting for more than four decades [1]. Conservative treatments, including lifestyle modification and pharmacotherapy, are often insufficient, particularly in cases of morbid or complicated obesity. In such situations, bariatric surgery remains the most effective approach for managing advanced obesity, enabling substantial and durable weight loss while improving or resolving many obesity-related comorbidities, such as type 2 diabetes, hypertension, and dyslipidemia [2].

Today, the most frequently performed procedures are sleeve gastrectomy (SG) and gastric bypass (GB), most commonly Roux-en-Y gastric bypass (RYGB). Less commonly used techniques include one-anastomosis gastric bypass (OAGB), single-anastomosis duodeno-ileal bypass with sleeve gastrectomy (SADI-S), and biliopancreatic diversion with duodenal switch (BPD-DS). The once-popular adjustable gastric band (AGB) is no longer recommended due to its complication profile and suboptimal bariatric effect [3,4].

Despite their high efficacy, bariatric procedures are not free from long-term nutritional complications. Gastric volume reduction and accelerated intestinal transit alter eating patterns, while bypass techniques additionally induce malabsorption. Consequently, many patients experience food intolerances—symptoms that occur after consuming specific foods (e.g., a sensation of food “sticking,” nausea, vomiting, bloating, diarrhea) and often lead to food avoidance. Observational studies indicate that roughly two-thirds of postoperative patients report such difficulties, which in some cases persist for several years [5].

Many poorly tolerated foods are difficult to digest (e.g., fried items) or of low nutritional value (e.g., confectionery), so their exclusion does not necessarily harm nutritional status [6]. However, intolerance of protein-, vitamin-, and mineral-rich foods may have more serious clinical consequences. For example, excluding meat can make it harder to meet recommended intakes of protein, iron, and vitamin B12, avoiding dairy may exacerbate calcium deficiency, and skipping vegetables predisposes individuals to insufficient fiber and vitamin intake [7]. Water intolerance can lead to dehydration and increased consumption of sugar-sweetened or artificially sweetened beverages [8]. Food intolerances may therefore contribute to malnutrition and nutrient deficiencies or, paradoxically, hinder weight loss and even lead to weight gain if poorly tolerated foods are replaced with highly caloric alternatives [9,10,11].

Despite growing interest, post-bariatric food intolerance remains poorly characterized, with studies differing in their definitions of intolerance, assessment tools, and time points.

This review aims to compile and discuss studies from the past ten years (2015–2025) that describe the prevalence and mechanisms of food intolerance after bariatric surgery and to present nutritional strategies for managing this problem. Special attention is given to intolerance of red meat, dairy products, water, bread, and vegetables. Understanding which foods cause the most difficulties—and why—will enable more personalized dietary advice, help prevent deficiencies, and support the long-term benefits of surgical obesity treatment.

## 2. Materials and Methods

A targeted narrative search of PubMed and the Cochrane Library was conducted covering the period from 1 January 2015 to 31 July 2025. Search strategies combined terminology for bariatric surgery procedures (e.g., “sleeve gastrectomy,” “Roux-en-Y gastric bypass,” “one-anastomosis gastric bypass,” “biliopancreatic diversion,” “SADI-S,” “banded RYGB”) with terms associated with food tolerance (e.g., “food intolerance,” “food tolerance,” “dietary tolerance,” “gastrointestinal symptoms,” “water intolerance”) to enhance the comprehensiveness of the search.

Studies were eligible if they included adult participants and reported tolerance to specific foods or food groups, or utilized validated tolerance assessment instruments, encompassing randomized controlled trials, prospective or retrospective cohort studies, cross-sectional studies, and systematic reviews. Studies were excluded if they were case reports or small case series (<10 participants), involved pediatric populations, lacked extractable tolerance outcomes, or were published in languages other than English. Titles and abstracts were independently screened for relevance, followed by full-text assessment for eligibility. Due to heterogeneity in outcome definitions, assessment instruments, and follow-up duration, data were synthesized narratively without meta-analysis.

## 3. Selected Food Intolerances After Bariatric Surgery

### 3.1. Meat Intolerance

Animal meat—particularly red meat, though in some patients also poultry—is frequently reported as difficult to ingest after bariatric surgery, with symptoms ranging from a “sticking” sensation and early satiety to nausea and vomiting even after small portions [12,13].

In the retrospective cohort study by Nicoletti et al. [13], 72 patients who underwent RYGB were included. Follow-up data were available for 63 patients at 1 year, 45 at 2 years, 56 at 3 years, and 41 at 4 years postoperatively. Red meat intolerance was reported in 49.2%, 42.2%, 46.4%, and 39% of patients at 1, 2, 3, and 4 years, respectively. After one year, patients with intolerance showed lower intake of calories, carbohydrates, and iron compared with tolerant patients. By the third year, weight regain was observed in the tolerant group (mean 2.9 ± 5.3 kg), whereas the intolerant group maintained stable body weight. No significant differences were identified between the groups in terms of clinical symptoms or biochemical parameters.

Boerlage et al. [5] conducted a cross-sectional study in which 249 patients were evaluated 2 years after RYGB using a bespoke questionnaire. Overall, 70.7% reported at least one food intolerance, with red meat being among the most frequent, cited by nearly 19% of respondents.

A randomized, triple-blind trial by Barstad et al. [14] included 109 patients (55 after SG, 54 after RYGB) assessed at 5 weeks and 12 months postoperatively. Food tolerance, measured with the Suter scale (Quality of Alimentation) [15] (0 = intolerance; 2 = full tolerance), was near maximal at baseline (1.8 and 1.9 for SG and RYGB, respectively). At 5 weeks, the score declined significantly in the SG group to 1.4, whereas after RYGB it remained higher at 1.7. At 12 months, partial improvement was observed. However, tolerance after SG (1.6) still remained lower than after RYGB (1.8).

In a prospective study using the Suter questionnaire, Gobato et al. [16] recorded an 80% prevalence of red meat intolerance in patients (*n* = 75) after banded RYGB (BRYGB), which intensified during the first postoperative year: good tolerance fell from 94.7% preoperatively to 33.3%, 28.0% and 20.0% at 3, 6 and 12 months, respectively.

In another prospective study using the same validated questionnaire, Pintor-de-la-Maza et al. [9] compared biliopancreatic diversion (BPD), gastric bypass, and sleeve gastrectomy in 66 patients. By 6 months, 87% of BPD patients ate red meat without difficulty, versus 41.2% and 61.9% in GB and SG. Differences persisted at 12 months (95% vs. 31.3% vs. 65.2%) and at 24 months, when full tolerance was reported by 100% after BPD but only 50% after GB and 73.3% after SG.

Díaz-Lara et al. [17] prospectively evaluated 65 patients after sleeve gastrectomy, assessing food tolerance at 1, 3, 6, 9 and 12 months using a modified Suter questionnaire. Tolerance to 59 specific food products was quantified on a five-point scale (0–4), and the probability of good tolerance was further estimated using a cumulative logit ordinal model (CLOM). A median score of at least 3 combined with a CLOM probability close to 80% was considered an indicator of good patient tolerance. While poultry (chicken and turkey) maintained stable median scores of 3 with probabilities of good tolerance around 74–75%, red meat consistently ranked among the least tolerated items. Lamb initially reached a median of 3 at month 1 (CLOM 57%) but declined thereafter, with scores falling to 2 and probability of good tolerance dropping to 39% by month 12. Veal and pork performed even worse: veal showed median values fluctuating between 2 and 3 with CLOM ranging from 40% at month 1 to 36% at month 12, while pork demonstrated the lowest tolerance, with median scores of 2–2.5 and CLOM decreasing steadily from 41% to 29% over follow-up.

In a prospective study of 93 SG patients, Ruiz-Tovar et al. [18] used the Suter questionnaire to assess food tolerance at 1 and 5 years postoperatively. Good tolerance of red meat was reported by nearly 80% of patients at 1 year, rising to about 90% at 5 years, indicating progressive improvement over time.

Long-term data from a cross-sectional analytic study by Cano-Valderrama et al. [19], who evaluated 161 patients a mean of 7.3 years after gastric bypass, mini-biliopancreatic diversion (MBPD), or malabsorptive duodenal switch (MDS), using the Suter questionnaire, showed that red meat remained the least accepted food category. In this cohort, 77% reported full tolerance, 17% consumed red meat with some discomfort, and 6% had eliminated it entirely. Intolerance rates did not differ between techniques, suggesting a shared pathophysiology among procedures that combine gastric restriction with varying degrees of malabsorption.

Procedure-specific factors may, however, modify outcomes, as illustrated by Figueiredo Reis et al. [20], who conducted a non-randomized prospective clinical trial comparing standard RYGB with banded RYGB. Among 94 patients assessed 6–24 months postoperatively using the Suter questionnaire, red meat tolerance differed markedly between groups. In the standard RYGB cohort, 68.3% reported being able to consume red meat easily, 38.6% experienced some difficulty, and only 4.3% reported complete intolerance. By contrast, in the BRYGB group, full tolerance was reported by just 31.7%, while 61.4% experienced difficulty and 14.9% reported complete avoidance of red meat. These differences were statistically significant (*p* = 0.001) and were accompanied by a lower median global food tolerance score in the BRYGB group (20 vs. 24 points), underscoring that the addition of a restrictive silastic ring impairs the ingestion of protein-rich solid foods such as red meat.

Meat intolerance in a substantial proportion of bariatric patients is multifactorial, involving an interplay of mechanical-digestive, hormonal–motility and sensory–psychological mechanisms [11]. The fibrous structure of meat requires prolonged chewing and proper initial digestion by stomach acid and pepsin. After creating a small gastric pouch, both the capacity of the stomach and the secretion of HCl are reduced, and consequently, the production of pepsin is diminished (Table 1). If patients swallow portions that are too large or insufficiently fragmented, meat fibers may stagnate in the pouch, provoking pressure, pain or nausea [21]. Studies also show that in the first months after RYGB surgery, red meat most often becomes a source of newly acquired taste aversion. Altered intestinal hormone profiles and rapid weight loss can modulate both taste perception and central food reward pathways, leading to aversion, and even a gag reflex, to the smell or taste of red meat [22].

Protein is a key nutrient after bariatric surgery, as it supports tissue repair, wound healing and the maintenance of lean body mass, which is responsible for resting metabolic rate and, consequently, proper weight loss. The recommended postoperative intake is 60–80 g/day or 1.1–1.5 g/kg ideal body weight [23]. Meat is a rich source of high-quality protein, iron and vitamin B12. Alternative protein-rich products include fish, eggs, legumes and dairy products [24]. For patients attempting to reintroduce meat, gradual strategies are recommended. These include initiating with finely minced or slow-cooked preparations (e.g., lean ground meat incorporated into steamed or stewed meatballs, or tender, slow-roasted cuts that disintegrate into fibers). Serving meat in minced form within soups, stews, or spreads may further enhance tolerance. Portions should be small (several dozen grams or less) and eaten slowly. Thorough chewing is essential: patients should spend more time than before on each bite and stop eating at the first sensation of fullness (Table 1) [25,26,27,28].

**Table 1:** Food Intolerances After Bariatric Surgery: Determinants and Dietary Management (compiled by the authors from [5,9,11,13,14,16,17,18,19,20,27,28,29]).

Food Products (Food Product Groups)	Determinants of Post-Bariatric Food Intolerance	Strategies for Improving Food Tolerance
Meat	Reduced stomach volume Reduced gastric juice secretion (hyposecretion)—decreased conversion of pepsinogen to pepsin, impaired protein digestion, and prolonged gastric retention Fibrous muscle structure Inadequate mastication of meat Swallowing of large boluses Disorders of taste and smell perception	Minced, tender meat—slow-cooked, steamed, or stewed until soft Serving in minced dishes (e.g., soups, meatballs, spreads) Consumption in very small portions Slow, thorough, and deliberate mastication Cessation of intake at the earliest indication of satiety Gradual reintroduction after symptom-free intervals
Dairy products	Unmasked lactose malabsorption following duodenum/jejunum bypass High osmolarity of milk-based products High fat content—delayed gastric emptying and symptom induction High sugar content in sweetened dairy products—contribution to bloating, flatulence, and dumping syndrome	Consumption of fermented, low-fat dairy products (e.g., yogurt, kefir, cottage cheese, skimmed milk) Use lactose-free products or lactase enzyme supplements Avoidance of high-sugar and high-fat dairy products (e.g., fruit yogurts) Limitation of ripened cheese consumption Replacement of sweetened yogurts with natural yogurts containing added fresh, cooked, or baked fruit
Raw leafy vegetables	Reduced stomach volume—excess of volume relative to the reduced gastric capacity High fiber content—delayed gastric emptying and increased risk of bloating Difficult mechanical breakdown during mastication—increased chewing effort and potential discomfort	Gradual expansion of the variety of vegetables consumed—starting with cooked, steamed, or roasted vegetables Gradual administration of raw vegetables—beginning with peeled, grated, finely diced, or blended forms Slow, thorough, and deliberate mastication
Cruciferous and allium vegetables	High content of fermentable fibers Presence of sulfur-containing compounds causing bloating and pain Increased colonic fermentation Potential increase in the risk of gastroesophageal reflux	Thorough cooking to soften fibrous structures Removal of tough parts and skins Chopping into small pieces and blanching—neutralization of sulfur compounds responsible for pungent taste and odor Administration in small amounts
Legumes	High content of fermentable fibers Presence of sulfur-containing compounds—promotion of bloating and abdominal pain Increased colonic fermentation Potential increase in the risk of gastroesophageal reflux	Administration in finely processed forms (e.g., blended or puréed, such as hummus, pastes) Prolonged cooking or soaking Administration in very small portions Gradual increase according to individual tolerance
Bread	Fluid absorption and swelling in the gastric pouch Formation of a dense (firm) bolus Dry consistency—provocation of the urge to drink during meals	Preference for toasted bread Chopping into small pieces and consumption in very small bites Thorough mastication Avoidance of drinking with meals Postponement of fresh, chewy bread
Pasta	Formation of a dense bolus—contribution to early satiety and abdominal discomfort	Consumption in very small portions Cooking until soft Preference for thin noodles over thick pasta Adherence to cooking time
Rice	Formation of a dense grain bolus—contribution to early satiety, discomfort, or nausea	Consumption in very small portions Thorough cooking until soft Adherence to cooking time Postponement and reattempt upon intolerance
Water	Impaired peristalsis in the gastric sleeve (aperistalsis) Postoperative edema—reduction in lumen Very low osmolarity and viscosity—delayed gastric emptying Rapid satiety or pain following minimal intake Gastric gurgling and distension	Frequent consumption of small sips of water between meals Preference for room temperature or mildly warm beverages Replacement with weak tea infusions (black, green, white, fruit, or herbal) Gradual reintroduction of still water

### 3.2. Dairy Intolerance

Dairy products, particularly milk and sweetened varieties, represent a frequent source of post-bariatric gastrointestinal discomfort, manifesting as bloating, abdominal pain, and diarrhea following consumption [29].

In a study by Boerlage et al. involving 249 participants two years after RYGB, 11.6% reported dairy intolerance compared with 4.4% in non-operated obese controls [5]. Westerink et al. [29] analyzed 168 individuals: 84 patients with morbid obesity scheduled for RYGB and 84 patients at least 12 months post-surgery. All participants underwent simultaneous hydrogen–methane breath testing (Lactose Breath Test, LBT) and oral lactose tolerance testing (Lactose Tolerance Test, LTT). Lactose malabsorption was defined as a positive result on at least one of these tests, whereas lactose intolerance was diagnosed when both LBT and LTT were positive in combination with significant gastrointestinal symptoms during testing. A questionnaire was also used to assess dairy consumption frequency and the occurrence of symptoms related to dairy intake in daily life. The study showed a significant increase in the incidence of lactose malabsorption after RYGB (17.9% vs. 29.8%), but this did not correspond to a proportional increase in clinically confirmed lactose intolerance (7.1% vs. 9.5%; *p* = 0.50). In contrast, subjective symptoms after dairy consumption in daily life were reported by 14.3% of pre-operative patients and 53.6% of post-operative patients. The most frequently reported triggers were milk (34.5%), ice cream (33.3%), and cream (29.8%). The authors concluded that, although RYGB more than doubles the risk of lactose malabsorption, this does not translate into a clear increase in the number of patients meeting the full clinical criteria for lactose intolerance.

In a study by Díaz-Lara et al. tolerance of dairy products varied mainly with fat content and degree of fermentation. As early as month 1, yogurt (both drinkable and set), skimmed milk, and cottage cheese achieved consistently high tolerance, with median scores of 3–4 and probabilities of good tolerance (CLOM) ranging from 78% to 91%. These values remained stable throughout the year, leading the authors to recommend them as a core, well-absorbed source of high-quality protein after SG. Cheese showed a different pattern: only cottage cheese consistently reached the “good” threshold (Me ≥ 3; CLOM ≥ 80%). Processed (melt) cheese also reached a median of 3, but with a lower probability (~68–69%), suggesting that its introduction should be conditional, especially in the early postoperative phase. Sliced and spreadable cheeses did not exceed a median of 3 until month 9 and were associated with CLOM values of 60–69%, supporting delayed, gradual reintroduction. By contrast, aged cheeses and full-fat milk maintained median scores ≤ 2 for most of the follow-up, with CLOM dropping below 60%. They were therefore classified as poorly tolerated and not recommended during the first postoperative year [17].

One mechanism underlying post-bariatric dairy intolerance is the unmasking of previously latent lactose digestion defects. After RYGB or BPD-DS, the duodenum and proximal jejunum—areas of highest lactase activity—are bypassed, impairing lactose hydrolysis (Table 1). Many patients who report “new” milk intolerance actually had mild lactase deficiency preoperatively, which is then exacerbated by surgical changes [28]. Westerink et al. suggest that the high prevalence of dairy-related complaints in their cohort likely reflects mechanisms beyond lactose itself, such as the relative difficulty digesting milk fat, altered gastrointestinal transit, or the hyperosmolar effect of milk-based drinks. These findings support the clinical principle that dyspeptic symptoms after dairy consumption warrant differential diagnosis: dietary elimination should be recommended only after objective confirmation of malabsorption, and in patients who remain symptomatic despite negative tests, reducing dietary fat content or switching to fermented dairy products may be beneficial [29].

From a nutritional standpoint, fermented, low-fat dairy products offer a favorable balance of high-quality protein and minimal gastrointestinal burden and should be prioritized in early dietary plans. Items with higher fat content or extensive processing—particularly hard and aged cheeses—are best introduced later or restricted altogether to prevent dyspeptic symptoms and optimize the utilization of the high-protein diet critical in the early postoperative period [17,27].

### 3.3. Vegetable Intolerance

Vegetables are a key source of fiber, vitamins, minerals, and phytochemicals. Therefore, their early and gradual reintroduction after bariatric surgery remains an important dietary goal. Typical symptoms of vegetable intolerance include a feeling of fullness, epigastric pain, bloating, and sometimes diarrhea—especially after consuming cruciferous, allium vegetables, legumes, raw leafy greens or stringy vegetables [17].

In the study by Pintor-de-la-Maza et al., vegetables were generally well accepted across all surgical techniques. At 6 months, “easy” tolerance was reported by 100% of patients after BPD, 88.2% after GB, and 90.5% after SG, with the remaining patients experiencing only minor difficulties. At 12 months, tolerance remained high (95.0%, 93.8%, and 87.0%, respectively), and by 24 months nearly all patients reported being able to consume vegetables without difficulty (94.1% after BPD, 91.7% after GB, and 100% after SG). In contrast, salads showed lower and more variable acceptance, particularly in the early postoperative period. At 6 months, full tolerance was reported by 95.7% of BPD patients, compared with only 64.7% after GB and 61.9% after SG (*p* = 0.017). In the same time frame, difficulties were noted in 4.3%, 23.5%, and 33.3%, while complete intolerance occurred in 0%, 11.8%, and 4.8% of patients, respectively. At 12 months, tolerance improved across all groups, with 90.0% of BPD, 62.5% of GB, and 82.6% of SG patients consuming salads without difficulty. Minor difficulties persisted in 5%, 25.0%, and 13.0%, and complete avoidance was reported in 5%, 12.5%, and 4.4% of patients, respectively. By 24 months, further improvement was observed, with 94.1% of BPD patients and 75.0% of both GB and SG patients reporting easy tolerance. Nonetheless, 5.9%, 8.3%, and 18.8% continued to experience difficulties, and complete intolerance persisted in 0%, 16.7%, and 6.2%, respectively [9].

In the study by Díaz-Lara et al., tolerance to vegetables after SG was heterogeneous and changed over time. Mushrooms, pumpkin, zucchini, chard, green beans, and spinach consistently achieved median tolerance scores of 3–3.5 (on a 0–4 scale of tolerance), with CLOM values ranging from 72% to 85%, and were therefore classified as recommendable. In contrast, tomatoes, cucumbers, and onions showed acceptable tolerance during the early months (median ~3; CLOM around 60%), but their acceptance progressively declined after month 6, reaching median values of 2 and CLOM estimates as low as 44–52% at 12 months, leading to their categorization as non-recommendable. Olives displayed a similar pattern, with CLOM dropping from 81% at baseline to 59% by one year. Celery showed the opposite trajectory, with initially poor acceptance (CLOM 52%) but gradual improvement to a median score of 3 and CLOM of 65% at the end of follow-up. Lettuce consistently exhibited the lowest tolerance, with median values ≤ 2–3 and a steady decline in CLOM to 41% at month 12, confirming its classification as non-recommendable. Among legumes, lentils were well tolerated throughout the year (Me 3; CLOM 77–82%) and classified as recommendable, while beans showed moderate improvement over time (CLOM 59–72%) and chickpeas progressed from poor early tolerance (CLOM 57%) to acceptable values (CLOM 70%) by the end of the first year, both being categorized as recommendable with caution [17].

Barstad et al. followed 109 patients (SG = 55; RYGB = 54) at 5 weeks and 12 months postoperatively. Acceptance of both leafy and cooked vegetables was the highest among all food categories evaluated: baseline mean scores on the Suter 0–2 scale were 1.9 in both cohorts, declining only marginally to 1.7 at week 5 and returning to 1.8 at month 12. Neither intra-group temporal changes nor inter-procedure comparisons reached statistical significance (*p* ≥ 0.44) [14].

In a study of 93 SG patients, Ruiz-Tovar et al. similarly found that vegetables, including lettuce, remained in the “easily tolerated” category at both one and five years postoperatively, indicating durable adaptation [18]. Two years after RYGB, only 4.8% of patients in the Boerlage cohort reported intolerance to lettuce and 9.6% intolerance to cruciferous vegetables, implying that more than 90% eventually tolerate most vegetables—contrasting with higher intolerance rates for red meat (≈19%) and confectionery (15–20%) in the same group [5].

Long-term data from Cano-Valderrama et al. support these findings: at a mean of 7.3 years post-surgery, ≥85% of patients (*n* = 161) across three surgical subgroups (RYGB, MBPD, MDS) consumed leafy and cooked vegetables without discomfort, and complete avoidance was rare. Such high acceptance suggests that restoration of gastrointestinal motility and enteric adaptation occur regardless of surgical modality, allowing vegetables to remain crucial for constipation prevention and micronutrient supply [19].

Reis et al. compared standard (*n* = 47) and banded RYGB (*n* = 47). Cooked vegetables were the best-tolerated category in both groups, with 100% and 93.6% of patients, respectively, reporting symptom-free consumption. Only three banded RYGB patients reported “some difficulty,” and the difference was not statistically significant (*p* = 0.242), indicating that even with a constrictive ring, thermally processed vegetables are largely well tolerated.

In contrast to vegetables, tolerance of salad showed greater variability between groups. After standard RYGB, 44 patients (93.6%) reported consuming salad without difficulty, 2 (4.3%) with some difficulty, and only 1 (2.1%) reported complete avoidance. Among BRYGB patients, however, full tolerance fell to 72.3%, while 11 (23.4%) experienced difficulties and 2 (4.3%) eliminated salad entirely. This difference was statistically significant (*p* = 0.001), suggesting that fibrous, bulky foods such as raw leafy vegetables may be particularly affected by the presence of the band [20].

Collectively, available evidence shows that vegetable tolerance tends to improve over time: after one to two years, most patients can ingest moderate portions of various vegetables without significant symptoms. Early intolerance is mainly attributed to the high fiber content of vegetables—small gastric capacity impairs mechanical breakdown, and bypassed intestinal segments limit digestion, leading to increased colonic fermentation (Table 1). To alleviate symptoms, patients are advised to cook or stew vegetables—thermal processing softens fibers, making them easier to break down and digest. Initially, vegetables with lower fiber content are best tolerated. Vegetables with seeds or tough fibers should be peeled or have their hard parts removed, and seeds discarded. Portion sizes should be small—the priority in a reduced stomach capacity is protein, so vegetables should serve as side dishes rather than taking up space needed for high-protein foods. If patients struggle even with cooked vegetables, introducing them in the form of purées or cream soups may be helpful. Gradual reintroduction of raw vegetables should follow a trial-and-error approach: start with small amounts (e.g., a few lettuce leaves or a slice of peeled tomato) and monitor reactions. Legumes are an excellent source of plant protein, but if patients have difficulty tolerating them, consuming them in blended forms (e.g., hummus or paste) may improve digestibility [26,27].

### 3.4. Cereal Products

Cereal products in various forms—bread, sliced bread, toasted bread, croutons, rice, noodles, pasta, oats, and others—often appear among foods that cause digestive problems [17,18]. In a study of 93 patients who underwent laparoscopic sleeve gastrectomy, Ruiz Tovar and colleagues showed that during the first postoperative year, bread, rice, and pasta constituted the group of foods causing the greatest digestion difficulties. Over time, tolerance improved markedly. Five years after SG, the overall score in the Suter Quality of Alimentation questionnaire (covering satisfaction, tolerance of eight food groups, and frequency of vomiting) rose from 21.3 to 24.2 points, and only a small number of patients still reported bread-related problems [18].

In the study by Díaz-Lara et al., tolerance of cereal products after SG was highly heterogeneous and product-dependent. Among breads, toast bread showed the most favorable profile, with median scores of 3 (on a 0–4 tolerance scale) already at the first month, and a steady increase in the probability of good tolerance (calculated using CLOM) from 60% at baseline to 82% at month 12, supporting its gradual introduction in the later recovery phase. White bread was less well accepted, with median scores fluctuating between 2.5 and 3 and CLOM values not exceeding 65%, thus being classified as recommendable with caution. In contrast, both croutons and sandwich-type sliced bread were consistently poorly tolerated, with median values around 2 and CLOM not surpassing 60% (41% for sliced bread at baseline). Regarding pasta and rice, noodles were the best tolerated, with stable median values of 3 and CLOM probabilities consistently above 75% throughout the follow-up period. By contrast, macaroni, spaghetti, and rice remained poorly tolerated, with median scores of approximately 2 and a progressive decline in CLOM (from 64% to 30% for macaroni, from 63% to 28% for spaghetti, and from 42% to 33% for rice across 12 months). Oats also demonstrated decreasing acceptance over time, with tolerance dropping from a CLOM of 67% at baseline to 41% at one year. Based on these findings, noodles and toast bread were classified as recommendable, white bread as recommendable with caution, whereas other pasta types, rice, oats, croutons, and sliced bread were placed in the non-recommendable category [17].

Pintor-de-la-Maza et al. evaluated food tolerance in a cohort of 66 patients undergoing biliopancreatic diversion, gastric bypass, or sleeve gastrectomy at 6, 12, and 24 months postoperatively, reporting marked differences between procedures in the acceptance of cereal products. At 6 months, full bread tolerance was observed in 95.7% of BPD patients, compared with only 35.3% after GB and 71.5% after SG (*p* < 0.001). This pattern persisted at 12 months (100% vs. 43.8% vs. 82.6%, respectively), while by 24 months the differences diminished, with universal tolerance in BPD and SG and 83.3% in GB (*p* = 0.06).

In contrast, tolerance of rice and pasta was consistently poorer. At 6 months, rice was consumed without difficulty by 95.7% of BPD patients, compared with 64.7% after GB and 81.0% after SG (*p* = 0.047). In the GB cohort, 17.6% reported some difficulties and 17.6% complete intolerance, while in SG 14.2% reported difficulties and 4.8% complete avoidance. Although tolerance improved modestly at 12 months (95.0% vs. 62.5% vs. 78.3%; *p* = 0.042), by 24 months a significant minority of GB and SG patients continued to experience intolerance (33.3% and 12.4% with difficulties, 0% and 6.3% complete avoidance, respectively). Pasta followed a similar trend: at 6 months, easy tolerance was achieved by 95.7% after BPD, but only 58.8% after GB and 66.7% after SG (*p* = 0.014), with complete intolerance reported in 17.6% and 9.5% of these groups, respectively. At 12 months, the proportion of patients tolerating pasta without difficulty rose to 100%, 62.5%, and 78.3% (*p* = 0.015), yet by 24 months, difficulties persisted in 41.7% of GB and 6.3% of SG patients, and complete intolerance remained in 12.4% of SG cases. Collectively, these findings indicate that while bread tolerance improves steadily after all procedures, rice and traditional pasta remain problematic for a considerable subset of GB and SG patients even two years postoperatively, whereas outcomes after BPD are consistently superior [9].

In a study by Cano-Valderrama et al., involving 161 patients with a mean follow-up of 7.3 years after gastric bypass, modified biliopancreatic diversion, or duodenal switch, bread was generally well tolerated: over 80% of participants reported consuming it without any associated symptoms, while fewer than 5% reported complete avoidance of bread products [19].

In the study by Figueiredo Reis et al., which compared 47 patients after standard RYGB with 47 after banded RYGB, bread was relatively well accepted in both groups. Following standard RYGB, 31 patients (66.0%) consumed bread without difficulty and 16 (34.0%) with some difficulty, while no patient reported complete avoidance. In the BRYGB group, only 24 patients (51.1%) tolerated bread easily, 21 (44.7%) experienced difficulties, and 2 (4.3%) reported total intolerance, though the difference was not statistically significant (*p* = 0.217). Rice was less well tolerated overall, and the presence of the band further impaired outcomes: 28 patients (59.6%) in the RYGB group reported good tolerance, 15 (31.9%) some difficulty, and 4 (8.5%) complete intolerance, compared with 19 (40.4%), 19 (40.4%) and 9 (19.1%), respectively, in the BRYGB group. Although differences did not reach statistical significance (*p* = 0.169), the data indicate a trend toward poorer acceptance of rice when a restrictive ring is used [20].

Barstad et al. reported that baseline bread tolerance scores, assessed on a 0–2 scale, were 1.8 for SG and 1.7 for RYGB. Five weeks postoperatively, these values decreased to 1.3 in both groups. By 12 months, an improvement was observed, with scores increasing to 1.5 for SG and 1.4 for RYGB. The differences between surgical techniques did not reach statistical significance (*p* ≥ 0.92). Rice followed a comparable pattern, with scores of 1.9 (SG) and 1.8 (RYGB) at baseline, declining to 1.4 and 1.7 at 5 weeks, and improving modestly to 1.5 and 1.7 after one year. Pasta tolerance similarly decreased from baseline values of 1.8 in both groups to 1.4 (SG) and 1.5 (RYGB) at 5 weeks, remaining low at 12 months (1.4 and 1.6, respectively). While between-group differences for bread and pasta were not significant, RYGB patients demonstrated a small but statistically significant advantage in rice tolerance over SG at one year (*p* = 0.018) [14].

Carbohydrate-rich cereal products such as bread, rice, and pasta are commonly associated with intolerance following bariatric surgery (Table 1). By absorbing fluid and swelling within the gastric pouch or sleeve, they may form a bulky, cohesive bolus that obstructs the narrowed outlet or anastomosis. Bread, in particular, poses additional challenges due to its dryness, which provokes a reflexive urge to drink—contrary to standard postoperative recommendations against fluid intake during meals. Rice and pasta can similarly clump together, resulting in early satiety, discomfort, or regurgitation. Furthermore, the insoluble fiber fraction of these foods may undergo colonic fermentation, contributing to bloating and gas. To mitigate these effects, dietary guidelines emphasize small portion sizes, slow and thorough mastication, and, where appropriate, the use of modified preparations (such as toasted bread) [26,27,28].

### 3.5. Water Intolerance

Maintaining adequate hydration after surgery is particularly important and constitutes one of the key criteria for the safe discharge of a patient from the hospital [11]. Dehydration has been identified as a common postoperative complication following bariatric surgery and is frequently cited as a reason for emergency department visits and hospital readmissions [8]. Moreover, sufficient fluid intake is essential for effective weight loss [30]. Unfortunately, some patients report difficulties with drinking water, especially during the early postoperative period. These difficulties may present as discomfort or even pain after a few sips, early satiety, nausea, or, in some cases, regurgitation of the ingested fluid—particularly when attempting to consume a larger volume at once.

In a study conducted by Elward et al., patients who had undergone LSG were evaluated for water and juice transit through the gastrointestinal tract using radiographic contrast studies performed at two time points: 48 h postoperatively and at three months. Imaging findings and patients’ self-reported difficulties with drinking water and juice indicated that water intolerance was significantly more common than juice intolerance. Specifically, 49% of patients reported difficulty drinking water 48 h postoperatively, with 29% still affected at three months. In contrast, difficulty consuming juice was considerably less prevalent, affecting only 7.8% of patients at the first time point and 5.9% at three months. The findings suggest that plain water—due to its lack of nutrients, electrolytes, and viscosity—is paradoxically less well tolerated than more complex liquids such as juice. The authors propose that the absence of osmolarity and peristalsis-stimulating properties in plain water may delay gastric emptying and lead to fluid retention in the proximal sleeve segment [11].

Water intolerance has also been observed after Roux-en-Y gastric bypass (RYGB), although less frequently. In a study by Boerlage et al., approximately 7.6% of patients two years post-RYGB reported an inability to tolerate plain water—either avoiding it entirely or experiencing discomfort upon consumption. Interestingly, this issue was not reported by any individuals in the control group, comprising obese patients who had not undergone surgery [5].

In a study by Díaz-Lara et al., tolerance to water after sleeve gastrectomy was suboptimal compared with other liquids. At month 1, median tolerance reached 3 (IQR 2–4) on the 0–4 scale, with a CLOM probability of good tolerance of only 78%. These values remained largely unchanged throughout the first postoperative year, with stable medians of 3 and CLOM probabilities gradually declining to 74% by month 12. The authors noted that cold water was more frequently associated with discomfort. Consequently, it was designated as “recommendable with caution” in the study’s dietary guidelines, in contrast to room-temperature water, which was classified as a fully recommended beverage.

In comparison to the moderate scores for water, significantly better tolerance was observed for broth, herbal infusions, and fruit juices. As early as the first postoperative month, these beverages achieved median scores of 3.0–3.5 points (up to 4.0 for broth in later months) and the highest CLOM probabilities among all fluid types (88–94%), which were maintained throughout the 12-month follow-up period. These findings support their unambiguous classification as recommended fluids after LSG. Coffee achieved a comparable median score (3 points), but with slightly lower, though still high, probabilities of good tolerance (79–84%). The authors therefore suggested that its intake should be individualized, particularly in patients reporting persistent upper gastrointestinal symptoms [17].

Postoperative water intolerance after bariatric surgery is multifactorial (Table 1). Following sleeve gastrectomy, resection of the distensible fundus and creation of a narrow, rigid “sleeve” abolish normal volume accommodation, whereas after Roux-en-Y gastric bypass the small pouch fills rapidly, amplifying early satiety and epigastric pressure. In both settings, the freshly operated stomach is oedematous and its capacity extremely limited, so that even a modest bolus of water can stretch the wall and provoke discomfort. Another explanation involves the differing physical properties of liquids: water is a Newtonian fluid with constant viscosity, whereas juices are non-Newtonian and more viscous. These rheological distinctions appear to affect gastric motility and flow dynamics, especially in the context of post-SG anatomical changes [11].

In the altered post-sleeve stomach, resection of the gastric pacemaker zone along the greater curvature can impair coordinated peristalsis, particularly in the vertical segment of the sleeve, which has been shown to be nearly motionless. In contrast, the antrum—if preserved—retains some contractile activity that may facilitate gastric emptying. However, its effectiveness in propelling low-viscosity fluids such as water appears diminished. Studies employing contrast-enhanced imaging have demonstrated that delayed fluid transit is associated with increased dysphagia and prolonged hospital stays. Moreover, both early and late recovery of esophageal motility, not gastric sleeve function, may play a key role in the gradual improvement of water tolerance. These findings suggest that post-SG water intolerance may stem from both altered gastric biomechanics and the unique physical characteristics of water, emphasizing the need for further investigation using dynamic imaging techniques and larger patient cohorts [11].

Management focuses on frequent, small sips with gradual increases in sip volume. Room-temperature water is preferred, and clear broths or diluted juices can be used as substitutes when plain water is poorly tolerated, as their modest osmolarity and nutrient content stimulate peristalsis. Successful hydration therefore requires both quantitative and qualitative fluid adjustments during the first postoperative months [27].

## 4. Conclusions

Food intolerance is a frequent yet under-recognized consequence of bariatric surgery, affecting long-term nutritional status and clinical outcomes. Symptoms are most pronounced early postoperatively but generally improve over time with physiological adaptation and patient-driven dietary modifications. Unmanaged intolerances can lead to micronutrient deficiencies, reduced quality of life, and suboptimal weight-loss maintenance. Effective management relies on comprehensive pre- and postoperative nutritional education, gradual food reintroduction, and regular multidisciplinary follow-up to personalize dietary guidance and optimize supplementation. Long-term success after bariatric surgery thus requires not only surgical expertise but also structured, patient-centered dietary management. Prospective studies with standardized definitions and objective outcome measures are warranted to further refine evidence-based postoperative guidelines.

## Data Availability

The original contributions presented in this study are included in this article. Further inquiries can be directed to the corresponding authors.

## Abbreviations

The following abbreviations are used in this manuscript:

AGB	Adjustable Gastric Band
BPD	Biliopancreatic Diversion
BS	Bariatric Surgery
BRYGB	Banded Roux-en-Y Gastric Bypass
CLOM	Cumulative Logit Ordinal Model
GB	Gastric Bypass
LSG	Laparoscopic Sleeve Gastrectomy
MBPD	Modified Biliopancreatic Diversion
MDS	Malabsorptive Duodenal Switch
OAGB	One Anastomosis Gastric Bypass
RYGB	Roux-en-Y Gastric Bypass
SADI-S	Single Anastomosis Duodeno-Ileal Bypass with Sleeve Gastrectomy
SG	Sleeve Gastrectomy
